# Identification of Male- and Female-Specific Olfaction Genes in Antennae of the Oriental Fruit Fly (*Bactrocera dorsalis*)

**DOI:** 10.1371/journal.pone.0147783

**Published:** 2016-02-04

**Authors:** Zhao Liu, Guy Smagghe, Zhongren Lei, Jin-Jun Wang

**Affiliations:** 1 Institute of Entomology, College of Plant Protection, Southwest University, Chongqing, 400715, China; 2 Institute of Plant Protection, Chinese Academy of Agricultural Sciences, Beijing, 100086, China; 3 Department of Crop Protection, Faculty of Bioscience Engineering, Ghent University, B-9000, Ghent, Belgium; Plant and Food Research, NEW ZEALAND

## Abstract

The oriental fruit fly (*Bactrocera dorsalis*) is a species of tephritid fruit fly, endemic to Southeast Asia but also introduced to many regions of the US, and it is one of the major pest species with a broad host range of cultivated and wild fruits. Although males of *B*. *dorsalis* respond strongly to methyl eugenol and this is used for monitoring and estimating populations, the molecular mechanism of the oriental fruit fly olfaction has not been elucidated yet. Therefore, in this project, using next generation sequencing technologies, we sequenced the transcriptome of the antennae of male and female adults of *B*. *dorsalis*. We identified a total of 20 candidate odorant binding proteins (OBPs), 5 candidate chemosensory proteins (CSPs), 35 candidate odorant receptors (ORs), 12 candidate ionotropic receptors (IRs) and 4 candidate sensory neuron membrane proteins (SNMPs). The sex-specific expression of these genes was determined and a subset of 9 OR genes was further characterized by qPCR with male and female antenna, head, thorax, abdomen, leg and wing samples. In the male antennae, 595 genes showed a higher expression, while 128 genes demonstrated a higher expression in the female antennae. Interestingly, 2 ORs (BdorOR13 and BdorOR14) were highly and specifically expressed in the antennae of males, and 4 ORs (BdorOR13, BdorOR16, BdorOR18 and BdorOR35) clustered with DmOR677, suggesting pheromone reception. We believe this study with these antennae-enriched OBPs, CSPs, ORs, IRs and SNMPs can play an important role in the detection of pheromones and general odorants, and so in turn our data improve our current understanding of insect olfaction at the molecular level and provide important information for disrupting the behavior of the oriental fruit fly using chemical communication methods.

## Introduction

The oriental fruit fly, *Bactrocera dorsalis*, is a tephritid fruit fly and one of the most devastating insect pests in South East Asia and in many Pacific Islands of the US, and it affects more than 150 different cultivated and wild fruits, including mango and guava [1, 2]. To date chemical management strategies are not suitable control methods because high levels of insecticide resistance have been developed in multiple regions of China [3, 4], and olfactory-mediated methods are preferred. Some chemicals originating from plants have been identified as attractants [5–7]. The best example being that males of *B*. *dorsalis* respond strongly to methyl eugenol and this is used for monitoring and estimating populations. But, although volatile traps have been used in many regions and an increasing number of volatiles with the potential to control *B*. *dorsalis* have been identified, the molecular mechanism of the oriental fruit fly olfaction has not been elucidated yet [8,9]. Therefore, more molecular knowledge on the different types of olfaction receptors in the antennae of *B*. *dorsalis* will contribute to the development of new strategies to control this pest using environment-friendly methods.

The first step toward understanding the mechanism of olfaction is to investigate the olfaction genes that encode the proteins that function in semiochemical detection. In recent decades, the identification of these genes and the study of their protein function have led to significant progress in understanding the molecular mechanisms of insect olfaction [10–13]. The insect olfactory system is highly selective and sensitive to semiochemicals and can discriminate specific odors to mediate important behaviors, including locating mates, food sources and oviposition sites. This function is achieved by the sensillae on the antennae, which detect the semiochemicals to activate olfactory sensory neurons and translate the chemical signals into nerve impulses to the brain. The odorant-binding proteins (OBPs), chemosensory proteins (CSPs), and sensory neuron membrane proteins (SNMPs) are involved in the perireceptor events, and the odorant receptors (ORs) and ionotropic receptors (IRs) play an important role in the signal transduction process [14]. OBPs that originate from the auxiliary cells and that are secreted into the aqueous sensillum lymph of the antenna (and/or maxillary palps) are thought to be the first proteins that bind with the semiochemicals to facilitate the transport of hydrophobic odorants through the aqueous surroundings to olfactory receptors [15,16]; some CSPs also function in this process [17,18]. After being released from the OBPs or CSPs, the semiochemicals are then transferred to the receptors on the olfactory sensory neuron dendritic membranes, ORs and/or IRs. ORs are seven-transmembrane-domain proteins that are coupled with co-receptors (Orcos) to form ion channels [19]. With the semiochemical (ligand) binding, the OR-Orco complexes are activated to trigger the signals that lead to the spikes generated in the brain [20–23]. IRs are derived from ionotropic glutamate receptors, which are expressed with the co-receptors IR8a and IR25a, and they function as chemosensory and gustatory receptors [24]. After the activation of the olfactory receptor, the semiochemicals are removed and degraded by the odorant-degrading enzymes (ODEs) [14, 25]. In addition to the OBPs, ORs, IRs and ODEs, other proteins, including SNMPs, aldehyde oxidases, aldehyde dehydrogenases, epoxide hydrolases, glutathione-S-transferases and cytochrome P450s, are involved in the olfactory process [14]. The SNMPs are expressed in the olfactory sensory neurons in the antennae; these proteins are hypothesized to play an important role in olfaction and gustation [26, 27].

The study of the molecular basis of olfaction remains in its infancy, although several *B*. *dorsalis* transcriptomes have been analyzed [8, 28–31]. Here we sequenced and annotated the antennal transcriptomes of males and females of *B*. *dorsalis* with the aim of identifying the genes encoding the proteins involved in olfaction. The sequencing generated a total of 55,968 unigenes, including candidate genes encoding CSPs, OBPs, ORs, IRs and SNMPs. Using qPCR experiments, we then validated the sex-specific expression for a selection of 9 OR genes. These improve our current understanding of insect olfaction at the molecular level for *B*. *dorsalis* and provide important information for disrupting the behavior of this important pest insect using environment-friendly chemical communication methods.

## Materials and Methods

### Ethics statement

No specific permits were required for the described field studies, and no specific permissions were required for these locations/activities. We confirm that these locations are not privately owned or protected in any way and that the field studies did not involve endangered or protected species.

### Insect rearing and total RNA extraction

A colony of the oriental fruit fly, which originated from Fujian, China, was reared on an artificial diet under standard conditions of 27°C, 70–80% relative humidity and a 14 h:10 h light:dark regime. The antennae were dissected from newly emerged adults (4-5-d-old; males and females were dissected separately), frozen in liquid nitrogen, and stored at -80°C until extraction. The male and female total RNA samples were separately isolated using an RNeasy Plus Micro Kit (Qiagen, Hilden, Germany) following the manufacturer’s instructions, and a gDNA eliminator spin column was used to remove the genomic DNA. The RNA quality was confirmed using a NanoVue UV-Vis spectrophotometer (GE Healthcare Bio-Science, Uppsala, Sweden), and the integrity was verified by agarose gel electrophoresis.

### Sequencing

The male and female cDNA libraries were separately constructed by Beijing Genomics Institute (BGI, Shenzhen, China) using an mRNA-Seq assay for paired-end transcriptome sequencing of the extracted RNA (20 μg from males and 22 μg from females). The poly (A) mRNA was purified by Oligo (dT) magnetic beads and further fragmented into short fragments in fragmentation buffer. The first-strand cDNA was synthesized using random hexamer primers, using the short mRNA fragments as templates. The second-strand cDNA was synthesized using dNTPs, RNase H buffer and DNA polymerase I. The synthesized short fragments were purified using a QIAquick PCR extraction kit (Qiagen). The sequencing adaptors were ligated after these short fragments were dissolved in ethidium bromide buffer (to end the repair process and to stop the single nucleotide A [adenine] addition). Fragments of a suitable size were selected as templates for PCR amplification. The constructed cDNA library was sequenced in the same manner, with single reads of 90 bp, using an Illumina HiSeq™ 2000 (BGI Tech Solutions, Hangzhou, China).

### Unigene assembly and functional annotation

After short and low-quality reads (the percentage of low-quality bases (base quality ≤ 10) is more than 20%) and adaptor sequences were removed, the Trinity program (release 20121005, BGI Tech., which combines three independent software modules, Inchworm, Chrysalis, and Butterfly, that are applied sequentially to process large volumes of RNA-seq reads) was used to assemble the clean read sequences from males and females into a single assembly, separately. Briefly, the process proceeds as follows:

Inchworm assembles the RNA-seq data into unique sequences of transcripts to generate full-length transcripts for a dominant isoform but then reports only the unique portions of alternatively spliced transcripts. Chrysalis clusters the Inchworm Contigs into clusters and constructs complete de Bruijn graphs for each cluster. Each cluster represents the full transcriptional complexity for a given gene (or sets of genes that share common sequences). Chrysalis then partitions the full read set among these disjointed graphs. Butterfly processes the individual graphs in parallel, tracing the paths that reads and pairs of reads take within the graph, ultimately reporting full-length transcripts for alternatively spliced isoforms and teasing apart transcripts that correspond to paralogous genes.

The resulting Trinity sequences are called unigenes. The unigenes are further processed for sequence splicing and redundancy removal using sequence clustering software (Tgicl v2.1) to acquire non-redundant unigenes that are as long as possible. Then, gene family clustering is used to divide the unigenes into two classes. One cluster is composed of several unigenes with more than 70% sequence similarity and named with the prefix C followed by the cluster id. The other clusters are singletons that are named with the prefix U followed by the gene id.

The identified unigenes were determined by comparing the sequences to databases, including Nr, Swiss-Prot, KEGG and COG, using the BLASTx algorithm with a significant cut-off E-value of < 10^−5^. The priority order of Nr, Swiss-Prot, KEGG and COG was employed to determine the sequence direction when the results from different databases conflicted. To compare the difference in the identified gene expression in the male and female transcriptomes, the FPKM (fragments per kb per million fragments) of genes in the male antennae transcriptome and female antennae transcriptome was compared. The Blast2GO program (v. 2.5.0 with default parameters, GO association performed by BLASTx against the NCBI NR database) was used for functional annotation of the gene from the antennal transcriptome. The open reading frames (ORFs) of the identified unigenes were predicted using the ORF Finder (http://www.ncbi.nlm.nih.gov/gorf/gorf.html) and verified based on the results of protein BLAST. The signal peptides of OBPs and CSPs were predicted by SignalP 4.0. The TMHMM server v. 2.0 (http://www.cbs.dtu.dk/services/TMHMM) was used to evaluate the transmembrane domains (TMDs) of the identified ORs. The phylogenetic tree was constructed based on the amino acid sequences of the candidate olfaction genes and on the amino acid sequences of the collected olfaction genes of other Diptera using neighbor joining and Poisson correction of distances in MEGA 5.0 software. The amino acid sequences were aligned using ClustalW. Node support was assessed using a bootstrap procedure based on 1000 replicates.

### Expression analysis of the candidate receptors by qPCR

To verify the expression profiles of the putative ORs in the different sexes and development stages, qPCR was performed using cDNAs prepared from male antennae, female antennae, heads (without antennae), thoraxes, abdomens, legs and wings on a Stratagene Mx3000P thermal cycler (Agilent Technologies, Wilmington, DE). Total RNA was extracted as described above. cDNA was synthesized using a First Strand cDNA Synthesis Kit (TaKaRa Biotechnology, Dalian, China), and it served as a template for qPCR. The PCR master mix (20 μl) contained 10 μl of iQ^TM^ SYBR Green Supermix (BIO-RAD, Hercules, CA), 1 μl of cDNA templates, 1 μl of each primer, and 7 μl of double-distilled water. Primers were designed using Primer 3 (http://bioinfo.ut.ee/primer3-0.4.0/) and are described in the supplementary materials (S1 Table). The following thermal program was executed: 95°C for 2 min, 95°C for 5 sec for 40 cycles, 60°C for 30 sec, and a final melting cycle (from 60 to 95°C). Three biological and two technical replicates were analyzed for each experiment. The expression level of each OR gene was quantified according to the 2^−ΔΔCT^ method using the α-tubulin gene as the control [32].

## Results

### Sequencing and unigene assembly

**Fig 1:**
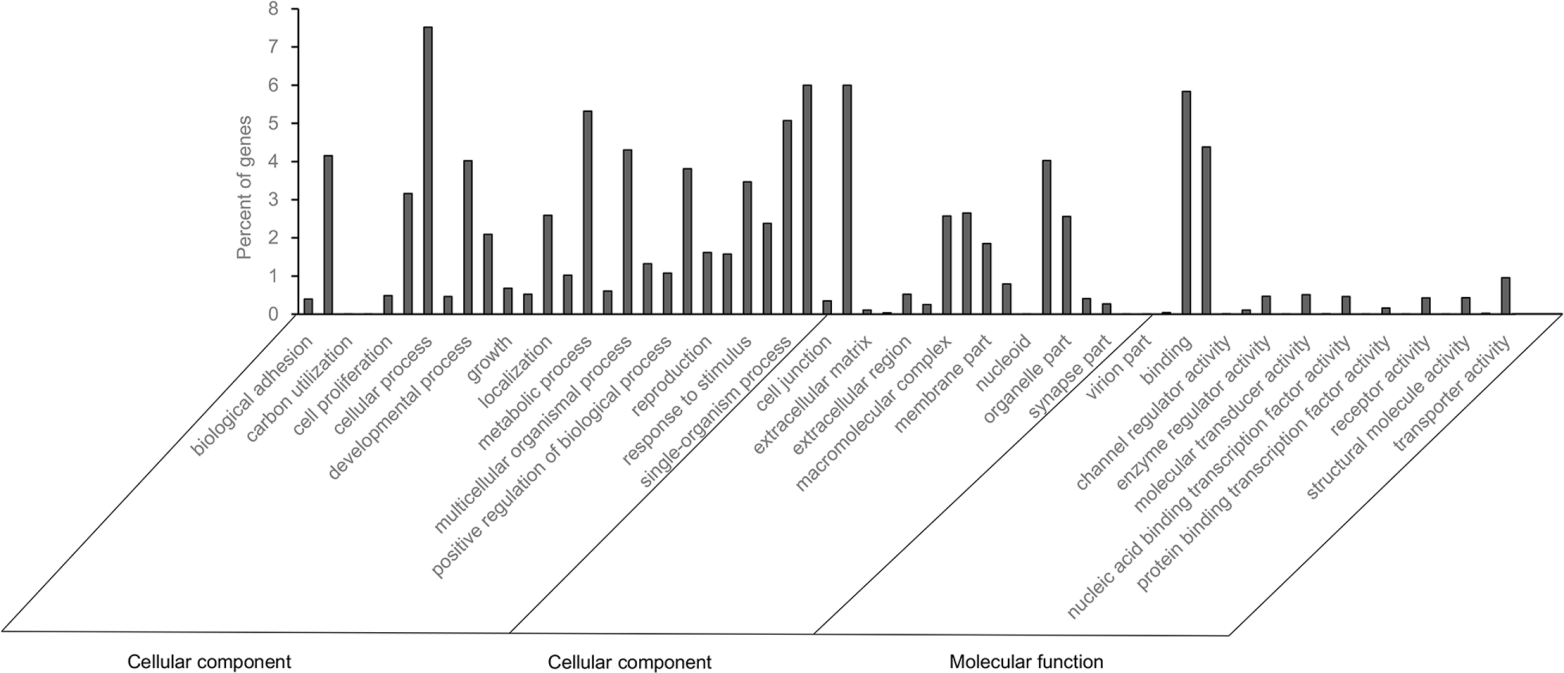
Distribution and comparison of male and female *B*. *dorsalis* unigenes annotated at GO level 2. The Y-axis shows the number of the sequence, the X-axis the areas of annotation and in each area, the sequences are further divided into subgroups at GO level 2.

Using the Illumina HiSeq 2000 sequencing system, 60,054,018 and 60,437,410 raw reads were obtained from the male and female samples, respectively (Submission ID: SUB803790, BioProject ID: PRJNA273535, in S1 Text). After removing low-quality, adaptor and contaminating sequence reads, male and female antennae yielded 51,611,382 and 53,906,604 clean reads, respectively. These clean reads were assembled into 103,615 contigs (N50 = 680) of an average length of 351 nt for male samples, and 121,663 contigs (N50 = 633) of an average length of 331 nt for female samples. All clean reads yielded 55,968 unigenes larger than 100 bp (N50 = 1,515) from male and female samples, with a mean length of 881 nt. Fig 1 shows the distribution of *B*. *dorsalis* unigenes in GO terms. Among these unigenes, 14,194 (25.36%) were assigned to a molecular function, 14,489 (25.89%) to a putative biological process, and 11,727 (20.95%) to a cellular component (Fig 1). Binding and catalytic activities were the most abundant GO terms in both the male and female sets, metabolic and cellular processes were the most represented GO terms in the biological process category, and the cell and membrane was the most represented GO terms in the cellular component category.

**Fig 2:**
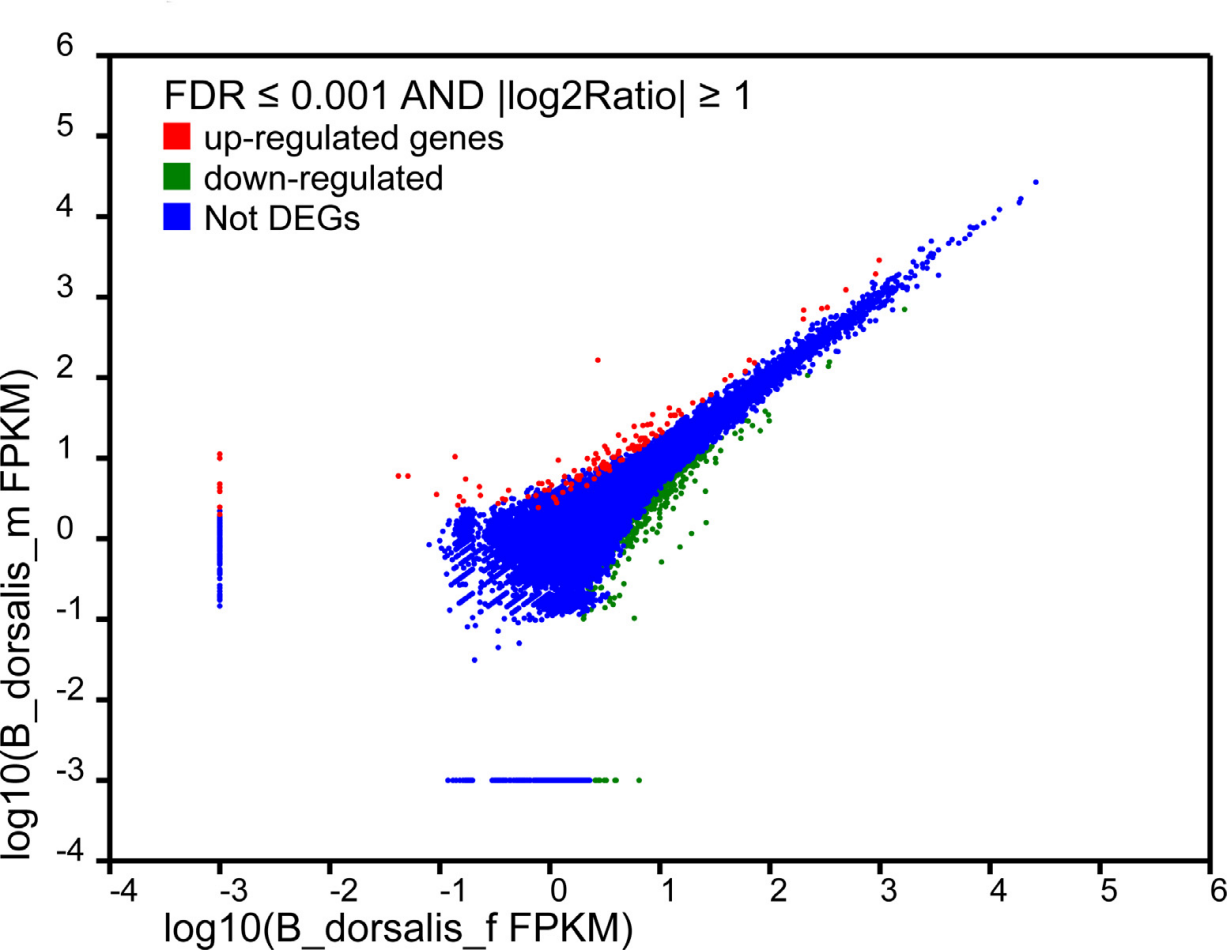
Statistical analysis of the differential expression of genes in the male (red) and female antennae (green) of *B*. *dorsalis*. If log2 (B_dorsalis_m_FPKM/B_dorsalis_f_FPKM) > 1, then this gene is more expressed in the male antennae, or if this log2<-1, then the gene shows a higher expression in the female antennae. In total, 595 genes showed a higher expression in the male antennae, and 128 genes were more expressed in the female antennae. B_dorsalis_m_FPKM denotes the FPKM of the selected gene in the male *B*. *dorsalis* antennae transcriptome, and B_dorsalis_f_FPKM denotes the FPKM of the selected gene in the female *B*. *dorsalis* antennae transcriptome.FDR: false discovery rate; FPKM: fragments per kb per million fragments; DEGs: different expressed genes.

The unigenes were searched against the NCBI non-redundant nucleotide database. Male antennae showed 595 genes with a higher expression, while 128 genes were more expressed in the female antennae (Fig 2). All candidate genes were compared using DNAMAN software (v. 8) to ensure their independence by removing shorter reads that overlapped with longer reads. All the amino acid sequences of the annotated olfactory genes are available in S2 Text.

### Identification of putative odorant-binding proteins

**Fig 3:**
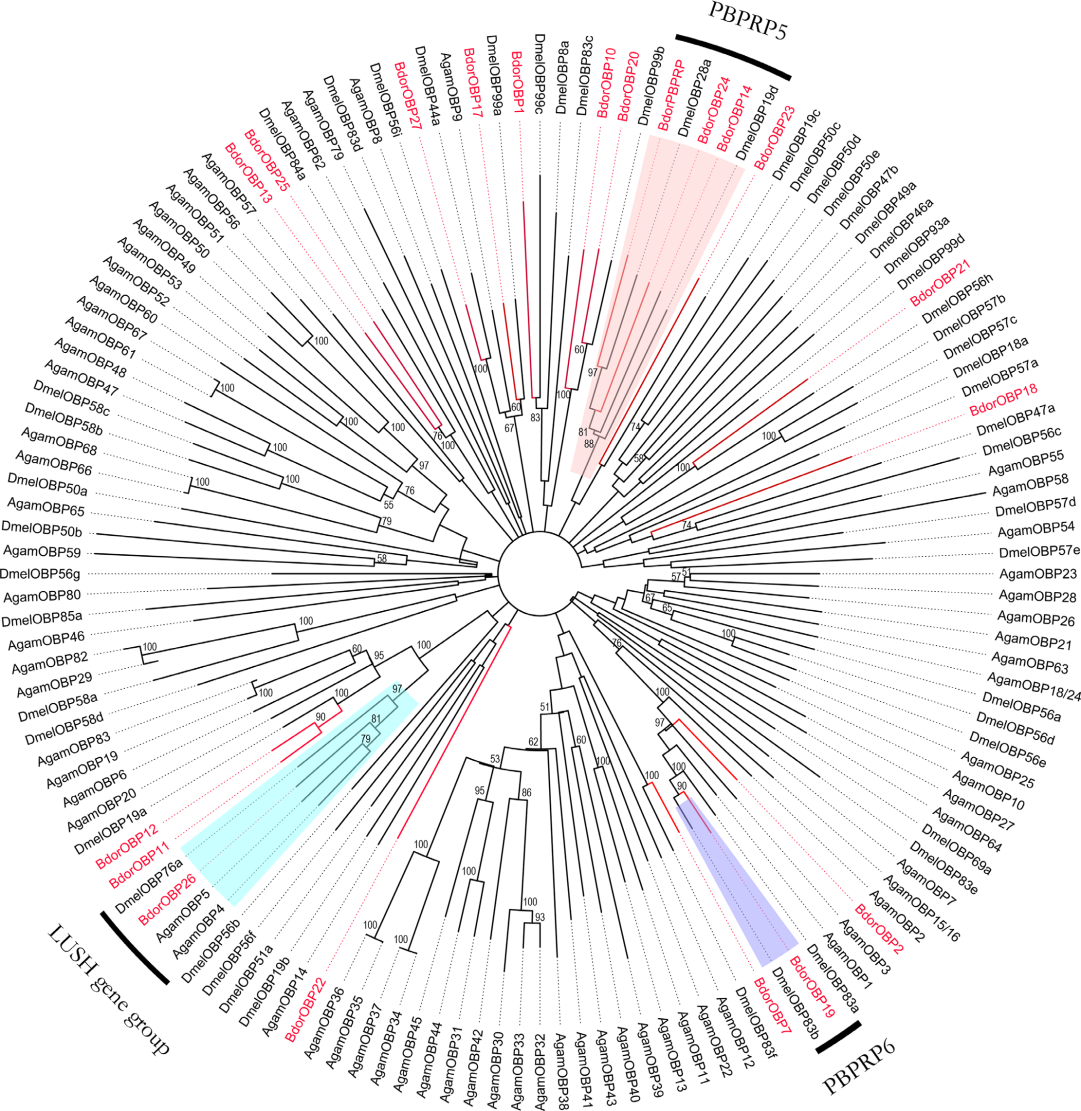
Phylogenetic relationships of the OBPs. Unrooted phylogenetic tree of the OBP protein sequences from *B*. *dorsalis*, *D*. *melanogaster* and *A*. *gambiae* constructed with MEGA 5.0.

In total, 20 candidate OBPs were identified from the *B*. *dorsalis* antennal transcriptome, including 15 unigenes with full-length ORFs and signal peptides, which were designated BdorOBPs. To compare the OBP families of *B*. *dorsalis* with those of other Diptera, the sequence similarities of the reported BdorOBPs to those of *Drosophila melanogaster* and *Anopheles gambiae* were determined [13, 14, 33–35], and a neighbor-joining tree was constructed (Fig 3). All putative OBPs were very similar to known OBPs from other Diptera, including signal peptides and highly conserved cysteines. The identified OBP genes encoded a diverse family of proteins and these genes clustered in different subgroups from the OBP genes of other Diptera species. A few closely related genes that clustered together with their counterparts from *D*. *melanogaster* were identified and named *Bdorobp14*, *Bdorobp22*, *Bdorobp24* and *Bdorpbprp*, respectively. Other *B*. *dorsalis* OBP genes clustered with at least one orthologous gene from Diptera. *Bdorobp26* clustered with the orthologous gene *Deml76a*, the *LUSH* gene, suggesting that this gene may have the same function in the perireceptor process (Fig 3).

### Identification of candidate chemosensory proteins

**Fig 4:**
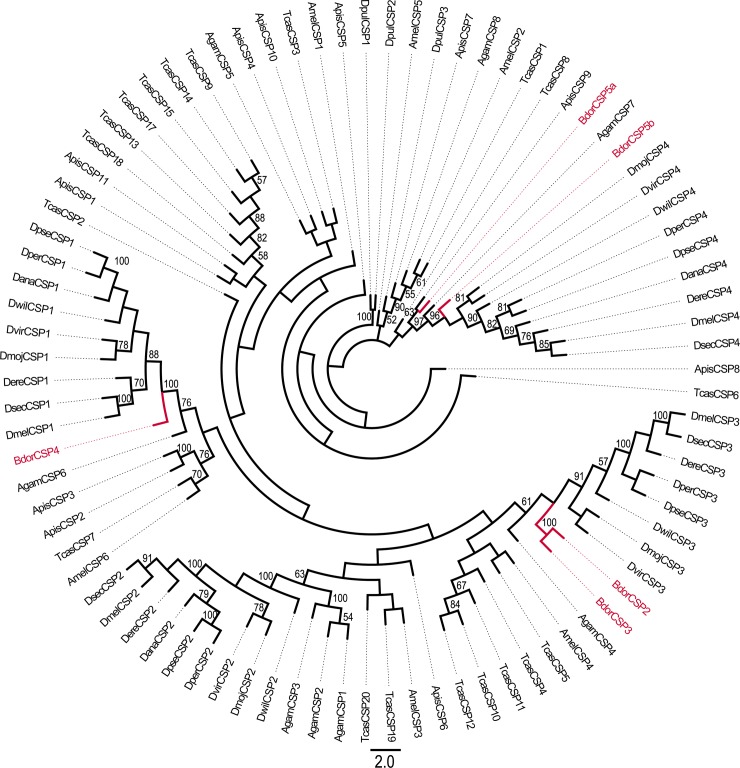
Phylogenetic relationships of the CSPs. Unrooted phylogenetic tree of CSP amino acid sequences from *B*. *dorsalis*, *D*. *melanogaster*, *A*. *gambiae*, *Ae*. *aegypti*, *Culex quinquefasciatus* and *Glossina morsitans morsitans*.

Five candidate CSPs were identified from the *B*. *dorsalis* antennal transcriptome, and three were predicted to have a full sequence with a signal peptide. The neighbor-joining tree revealed that all five sequences clustered with one orthologous gene and that the candidate CSPs could be easily identified (Fig 4). All BdorCSPs were very similar to the CSP family of other Diptera species. The unigene *Bdorcsp1* was predicted to have the same function as its paralog, *Bdorcsp2*, in feeding and oviposition [18]. The identified CSPs from *B*. *dorsalis* were numbered BdorCSP1-5; the information on the CSPs is provided in Table 1.

**Table 1:** Unigenes of candidate odorant-binding proteins and chemosensory proteins.

	Gene name	Length (amino acids)	Peptide signal	Full length	C nb	E-value	BLASTx best hit (Reference/Name/species)
Chemosensory proteins							
U15847	BdorCSP1	158	yes	Yes	5	3.0E-45	gi|281426843| chemosensory protein 2 [*Glossina morsitans morsitans*]
U15925	BdorCSP2	120	yes	Yes	3	4.0E-78	gi|171903815| chemosensory protein [*Bactrocera dorsalis*]
C6798C1	BdorCSP3	157	yes	Yes	4	3.0E-45	gi|281426847| chemosensory protein 4 [*Glossina morsitans morsitans*]
U2213	BdorCSP4	111	yes	no	7	3.0E-38	gi|281426849| chemosensory protein 5 [*Glossina morsitans morsitans*]
C5409C1	BdorCSP5	44	no	no	0	4.0E-16	gi|281426849| chemosensory protein 5 [*Glossina morsitans morsitans*]
Odorant-binding proteins							
U10931	BdorOBP7	142	yes	yes	7	1E-45	gi|198132065| odorant-binding protein 83g [*Drosophila pseudoobscura*]
U12568	BdorOBP11	92	no	no	4	1E-30	gi|291088326| odorant-binding protein 5 [*Delia antiqua]*
U13232	BdorPBPRP	146	yes	yes	7	5E-33	gi|68067701| Pheromone-binding protein-related protein 5 [*Drosophila melanogaster*]
U17149	BdorOBP12	148	yes	yes	6	1E-42	gi|291088326| odorant-binding protein 5 [*Delia antiqua*]
U18889	BdorOBP13	167	yes	yes	7	5E-29	gi|194741516| odorant-binding protein 84a [*Drosophila pseudoobscura*]
U19265	BdorOBP14	145	yes	yes	7	1E-16	gi|194893499| odorant-binding protein 19d [*Drosophila melanogaster*]
U22175	BdorOBP10	107	no	no	4	9E-16	gi|281426837| odorant-binding protein 21 [*Glossina morsitans morsitans*]
U22813	BdorOBP1	159	yes	yes	6	4E-14	gi|281426839| odorant-binding protein 22 [*Glossina morsitans morsitans*]
U22908	BdorOBP17	151	yes	yes	7	2E-32	gi|281426799| odorant-binding protein 2 [Glossina morsitans morsitans]
U26215	BdorOBP18	116	no	no	6	6E-12	gi|198458811| odorant-binding protein 57c [*Drosophila pseudoobscura*]
C389C1	BdorOBP19	155	yes	yes	6	2E-56	gi|291088322| odorant-binding protein 3 [*Delia antiqua*]
C3324C1	BdorOBP20	142	yes	no	5	8E-35	gi|195158839| odorant-binding protein 21 [*Glossina morsitans morsitans*]
C5896C1	BdorOBP21	136	yes	yes	7	2E-21	gi|226823099| odorant-binding protein 56h [*Drosophila melanogaster*]
C6728C1	BdorOBP22	157	yes	yes	6	5E-07	gi|268056761| odorant-binding protein 19d *[Drosophila melanogaster*]
U451	BdorOBP23	105	no	no	5	5E-12	gi|281426805| odorant-binding protein 5 [*Glossina morsitans morsitans*]
U1624	BdorOBP24	141	yes	yes	9	1E-20	gi|195432795| odorant-binding protein 1 [*Delia antiqua*]
U1841	BdorOBP25	170	yes	yes	9	7E-27	gi|195395570| odorant-binding protein 84a [*Drosophila pseudoobscura*]
U4042	BdorOBP26	123	yes	yes	6	1E-43	gi|226823103| odorant-binding protein 7 [*Delia antiqua*]
U6328	BdorOBP2	148	Yes	yes	6	1E-84	gi|171903817| odorant-binding protein [*Bactrocera dorsalis*]
U5460	BdorOBP27	148	yes	yes	6	3E-56	gi|289742907| odorant-binding protein *[Glossina morsitans morsitans*]

### Identification of olfactory receptor proteins

**Fig 5:**
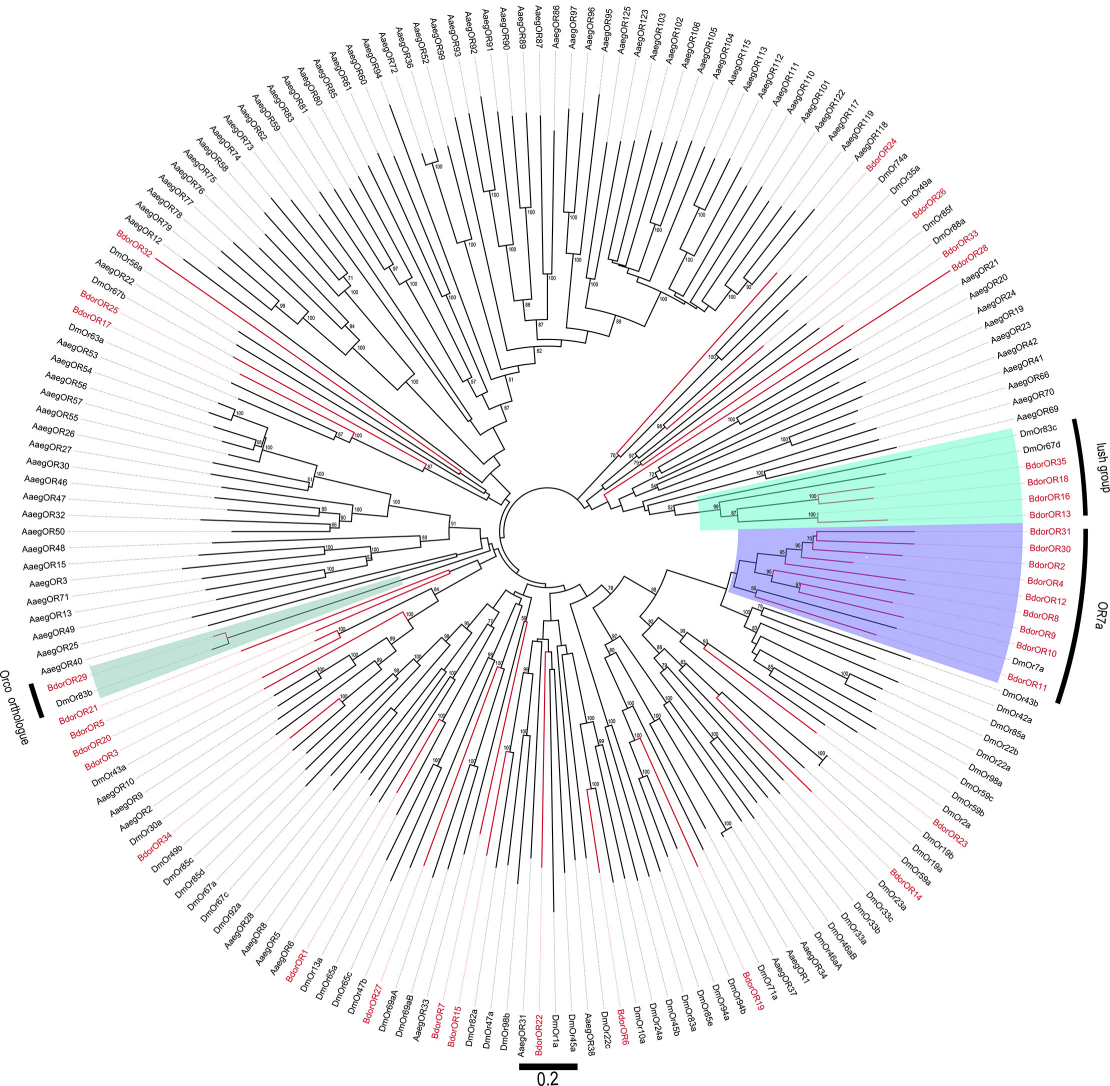
Phylogenetic relationships of the ORs. Unrooted phylogenetic tree of OR protein sequences from *B*. *dorsalis*, *D*. *melanogaster* and *A*. *gambiae*.

In total, 35 OR candidate genes were identified in the analysis of the *B*. *dorsalis* transcriptome and numbered BdorOR1-35. Eighteen sequences encoded complete proteins (BdorOR6, BdorOR29, BdorOR3, BdorOR9, BdorOR11, BdorOR13, BdorOR15, BdorOR17, BdorOR18, BdorOR19, BdorOR21, BdorOR22, BdorOR25, BdorOR27, BdorOR28, BdorOR34, and BdorOR35) that were predicted to contain 6 or 7 transmembrane domains. The other BdorOR proteins were predicted to contain 2–6 transmembrane domains. Most OR candidate genes clustered with at least one orthologous gene in the phylogenetic tree (Fig 5). One co-receptor, BdorOR29, was identified because of its high similarity with DmOR83b. Four ORs (BdorOR13, BdorOR16, BdorOR18, BdorOR35) clustered with DmOR67d, the cVA receptor in *Drosophila* (lush group), suggesting that these ORs might be involved in pheromone reception in *B*. *dorsalis*. Nine ORs were classified into another subgroup and shared high similarity with DmOR7a. Information, including the unigene reference, length, and BLASTx best hit, of all ORs is provided in Table 2.

**Table 2:** Unigenes of candidate olfactory receptors and inotropic receptors.

Reference	Name	Length (amino acids)	Full length	TMD No	E-value	Blast p hit
Olfactory Receptors						
C739C1	BdorOR1	326	no	4	1E-81	gb|EDY70605.1| Or13a [Drosophila pseudoobscura]
C788C3	BdorOR2	409	no	4	6E-52	gb|AAF59173.2| odorant receptor 43b [Drosophila melanogaster]
C3544C1	BdorOR3	403	no	6	1E-44	gb|ACH69149.1| odorant receptor 2 [Anopheles stephensi]
C3971C1	BdorOR4	265	no	2	8E-42	gb|ADD14625.1| olfactory receptor Or43b [Drosophila mauritiana]
C5339C2	BdorOR5	173	no	2	1E-15	gb|ADF42902.1| odorant receptor 10 [Culex quinquefasciatus]
C4154C1	BdorOR6	400	yes	8	2E-113	gb|EDY72807.1| Or10a [Drosophila pseudoobscura]
U3948	BdorOR7	340	no	5	9E-80	gb|EDY73962.1| odorant receptor N [Drosophila pseudoobscura]
U4218	BdorOR8	414	no	4	9E-49	gb|ADK48026.1| odorant receptor 42b [Drosophila melanogaster]
U14846	BdorOR9	400	yes	7	5E-69	gb|ADK48091.1| odorant receptor 7a [Drosophila melanogaster]
U15921	BdorOR10	399	no	4	1E-73	gb|ADK48089.1| odorant receptor 7a [Drosophila melanogaster]
U16745	BdorOR11	454	no	6	1E-73	gb|ADK48058.1| odorant receptor 7a [Drosophila melanogaster]
C1686C3	BdorOR12	378	no	4	2E-53	gb|ADK48091.1| odorant receptor 7a [Drosophila melanogaster]
U33	BdorOR13	394	no	6	6E-48	gb|AAF50161.3| odorant receptor 67d [Drosophila melanogaster]
U350	BdorOR14	342	no	5	1E-75	gb|AAF47004.1| odorant receptor 59a [Drosophila melanogaster]
U803	BdorOR15	406		6	5E-92	gb|EAL28773.3| Or82a [Drosophila pseudoobscura]
U1586	BdorOR16	142	no	2	3E-27	gb|ADG96063.1| putative odorant receptor [Stomoxys calcitrans]
U1859	BdorOR17	403		6	8E-102	emb|CBW30605.1| odorant receptor [Drosophila simulans]
U3061	BdorOR18	406		6	2E-58	gb|AAF50161.3| odorant receptor 67d [Drosophila melanogaster]
U3077	BdorOR19	423	yes	8	3E-83	gb|EAL27244.2| Or94a [Drosophila pseudoobscura]
U3141	BdorOR20	117	no	2	1E-20	gb|EAL24773.2| Or43a [Drosophila pseudoobscura]
C173C1	BdorOR21	415		6	9E-38	ref|XP_001662355.1| odorant receptor [Aedes aegypti]
U8871	BdorOR22	368	Yes	6	7E-48	gb|EAL25642.2| Or45a [Drosophila pseudoobscura]
U8910	BdorOR23	401	Yes	5	1E-68	gb|AAF45759.1| odorant receptor 2a [Drosophila melanogaster]
U10148	BdorOR24	417	Yes	5	1E-28	gb|AEB91834.1| odorant receptor 35a [Drosophila melanogaster]
U11167	BdorOR25	417	Yes	6	1E-80	emb|CBW30605.1| odorant receptor [Drosophila simulans]
U11993	BdorOR26	319	no	4	4E-126	gb|ABW80750.1| odorant receptor [Rhagoletis suavis]
U12022	BdorOR27	423	Yes	6	1E-40	emb|CBW30763.1| odorant receptor [Drosophila pseudoobscura]
U13527	BdorOR28	456	Yes	6	3E-76	gb|ADK48358.1| odorant receptor 43a [Drosophila melanogaster]
CL538C2	BdorOR29	473	Yes	7	0	gb|ADK97803.1| odorant receptor Or83b [Bactrocera cucurbitae]
C2326C1	BdorOR30	400	no	4	1E-54	gb|ADK48089.1| odorant receptor 7a [Drosophila melanogaster]
C2460C2	BdorOR31	392	no	4	6E-55	gb|ADK48091.1| odorant receptor 7a [Drosophila melanogaster]
C4824C1	BdorOR32	186	no	3	6E-07	gb|ADF42902.1| odorant receptor 10 [Culex quinquefasciatus]
C5300C1	BdorOR33	427	no	3	3.E-34	gb|AAF55018.2| odorant receptor 88a [Drosophila melanogaster]
C6087C2	BdorOR34	371	Yes	6	2E-136	gb|EAL26214.1| Or49b [Drosophila pseudoobscura]
C8295C1	BdorOR35	396	Yes	6	1.E-59	gb|AAF50161.3| odorant receptor 67d [Drosophila melanogaster]
Ionotropic Receptors						
C2923C2	BdorIR1	719	no	2	1E-21	gb|AAF55757.3| ionotropic receptor 92a [Drosophila melanogaster]
U23089	BdorIR2	78	no	0	9E-38	gb|AAF51569.2| ionotropic receptor 21a [Drosophila melanogaster]
U31214	BdorIR3	76	no	0	3E-34	gb|EAT39625.1| glutamate receptor, ionotropic, n-methyl d-aspartate [Aedes aegypti]
C1154C3	BdorIR4	660	no	4	0	gb|AAF49071.1| ionotropic receptor 76b [Drosophila melanogaster]
U7132	BdorIR5	887	no	3	3E-10	gb|ADU79035.1| ionotropic receptor 64a [Drosophila melanogaster]
U7187	BdorIR6	521	no	4	4E-45	gb|AAF56134.4| ionotropic receptor 94e [Drosophila melanogaster]
U9427	BdorIR7	822	no	3	0	gb|AAF57255.3| ionotropic receptor 40a [Drosophila melanogaster]
U14774	BdorIR8	638	no	3	4E-154	gb|ADQ74919.1| ionotropic receptor 75a [Drosophila melanogaster]
U16570	BdorIR9	737	no	3	0	gb|AAF55817.2| ionotropic receptor 93a, isoform A [Drosophila melanogaster]
U23532	BdorIR11	172	no	0	3E-48	gb|EDS27909.1| ionotropic glutamate receptor subunit ia [Culex quinquefasciatus]
U5750	BdorIR13	113	no	1	4E-43	gb|ABI31184.1| glutamate receptor IIE [Drosophila melanogaster]
U14553	BdorIR14	261	no	1	1E-80	gb|ABI31184.1| glutamate receptor IIE [Drosophila melanogaster]
C8433C2	BdorIR15	944	no	3	0	gb|AAF46470.2| ionotropic receptor 8a [Drosophila melanogaster]
U215	BdorIR16	940	no	3	0	gi|401063649|gb|AFP89966.1| ionotropic receptor 25a [Musca domestica]

### Identification of candidate ionotropic receptors

**Fig 6:**
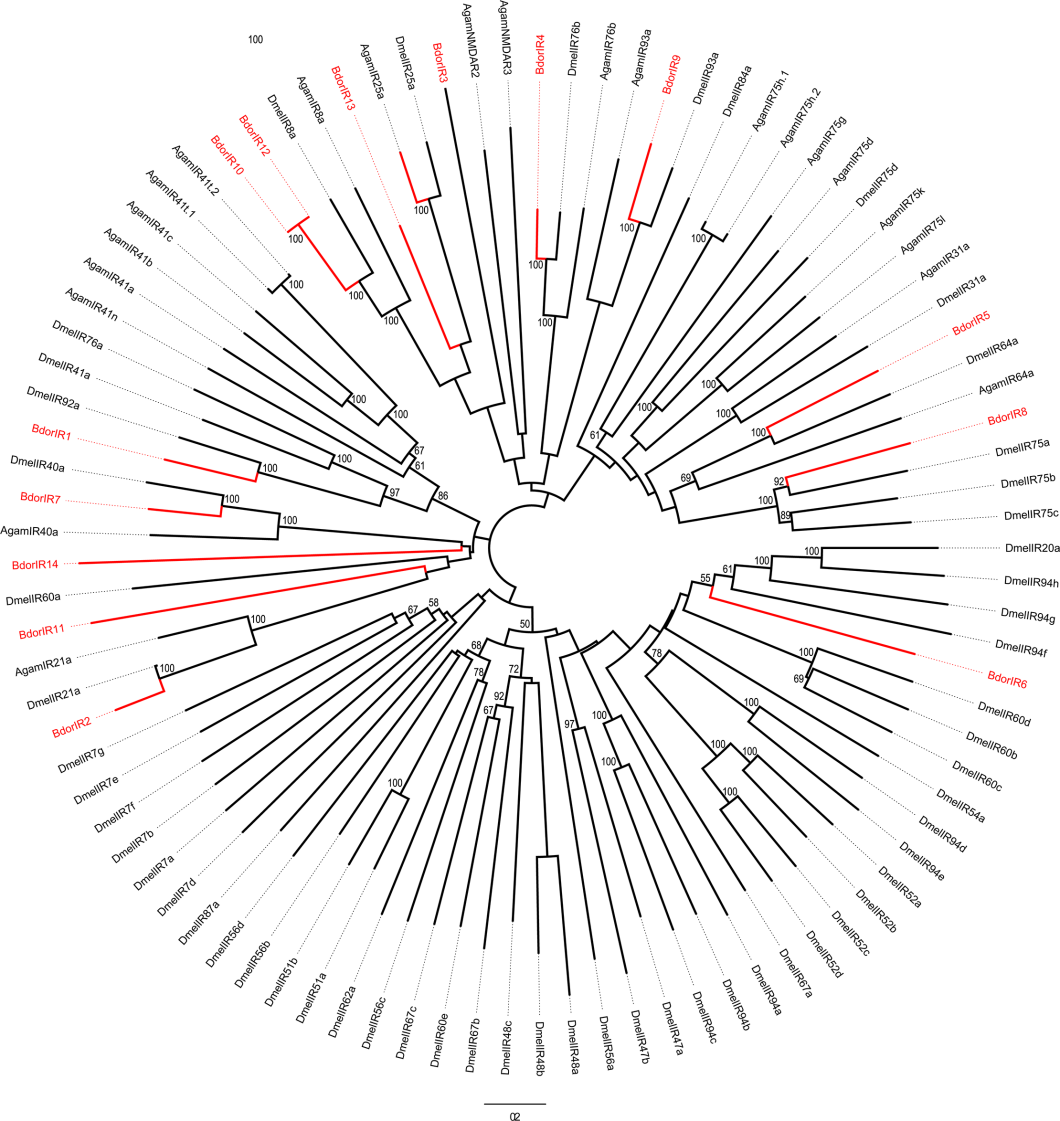
Phylogenetic relationships of the IRs. Unrooted phylogenetic tree of IR protein sequences from *B*. *dorsalis*, *D*. *melanogaster* and *A*. *gambiae*.

The IR sequences in the *B*. *dorsalis* antennal transcriptome are shown based on their similarity with identified insect IRs. Twelve candidate IR sequences were identified in the antennal transcriptome analysis, and three of them possessed at least three transmembrane domains as predicted by TMHMM 2.0. An un-rooted phylogenetic tree was constructed to evaluate the relationship among the IRs from *B*. *dorsalis*, *D*. *melanogaster* and *A*. *gambiae* (Fig 6). Most of the IR candidate genes clustered with at least one orthologous gene in the phylogenetic tree. The names, unigene references, lengths, and best BLASTx hits of all 14 IRs are presented in Table 2.

### Identification of candidate sensory neuron membrane proteins

In *D*. *melanogaster*, two SNMPs have been identified and confirmed to play important roles in pheromone detection. Analysis of the *B*. *dorsalis* transcriptome resulted in the identification of four SNMP genes based on the best hit; these genes were designated *Bdorsnmp1a*, *Bdorsnmp1b*, *Bdorsnmp2a* and *Bdorsnmp2b*. The nucleotide sequences of the SNMPs are listed in the supplementary material (S2 Text). BdorSNMP1a and BdorSNMP2a were predicted to have complete sequences containing two transmembrane domains. SNMP1b and SNMP2b are partial sequences; SNMP2b contains only one transmembrane domain, and no transmembrane domain is present in SNMP1b.

### Tissue- and sex-specific expression of candidate OR genes

**Fig 7:**
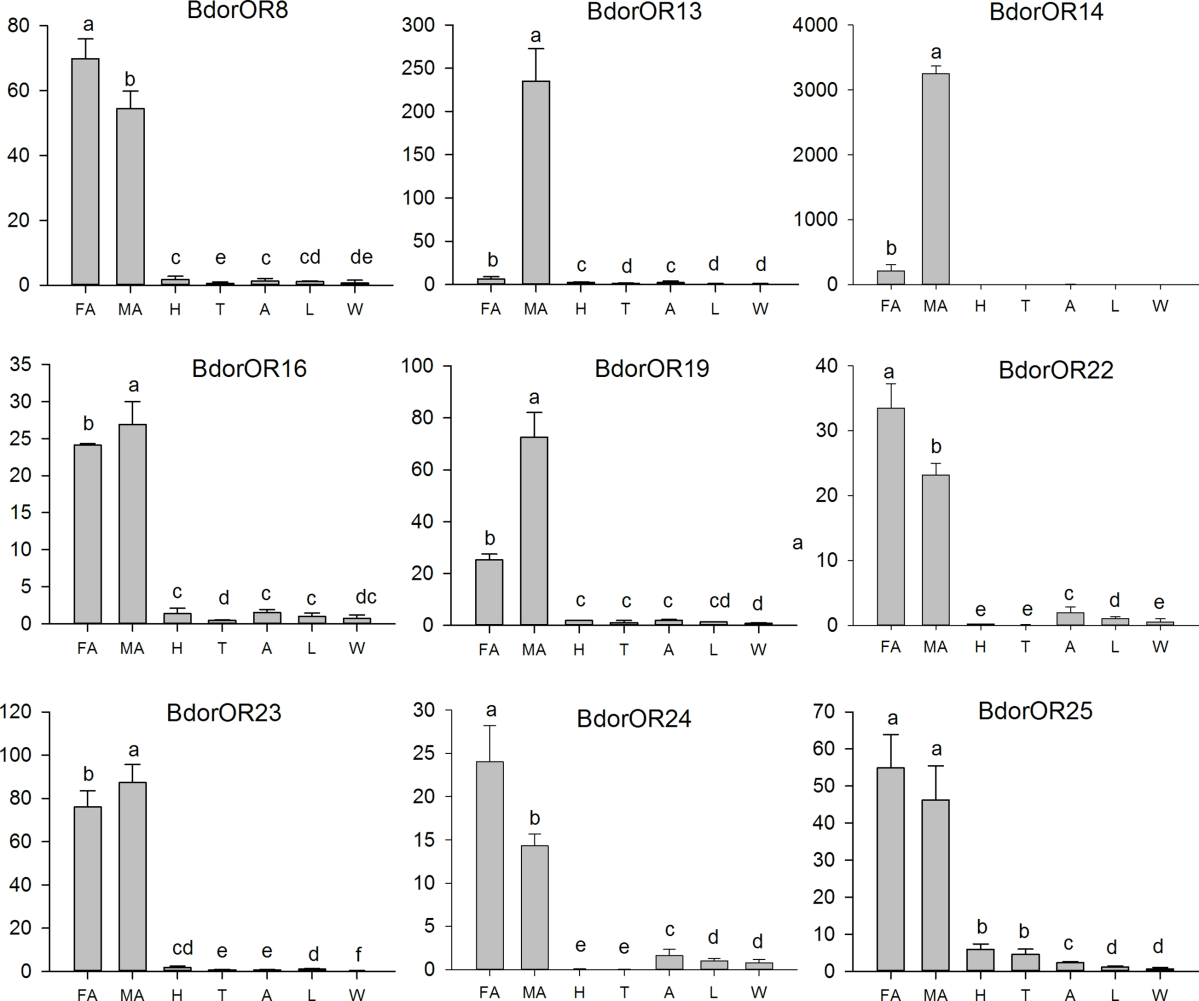
Tissue- and sex-specific expression patterns of candidate *B*. *dorsalis* ORs. The *BdorOR13* and *BdorOR14* genes are highly and specifically expressed in the male antennae, and also *BdorOR19* shows a 3-fold higher expression in the male antennae. The candidate genes *BdorOR8*, *BdorOR16*, *BdorOR22*, *BdorOR23*, *BdorOR24* and *BdorOR25* exhibit a high expression in both the male and female antennae, in contrast to a (very) weak expression in other tissues. The Y-axis represents the relative expression level and the X-axis the different tissues of *B*. *dorsalis*. FA: female antennae; MA: male antennae; H: head (without antennae); T: thorax; A: abdomen; L: legs; W: wings. Error bars represent the standard error of the measurement. An asterisk denotes statistically significant differences (p<0.05) between gene expression in the male and female antennae; the statistical analysis was carried out by SPSS 16 software (*t*-test).

The expression patterns of the candidate ORs in male antennae, female antennae, heads (without antennae), thoraxes, abdomens, legs and wings were analyzed by qPCR. All examined ORs were detected in *B*. *dorsalis* antennae, and some of the ORs were weakly expressed in other parts of the body (Fig 7). Interestingly, two OR genes, *Bdoror13* and *Bdoror14*, were expressed highly and specifically in the male antennae. In detail, the respective expression level of *Bdoror13* and *Bdoror14* was 235 and 3254 times over the reference gene and this corresponds to 37 and 15 times higher in the male antennae than in the female ones. The *Bdoror19* gene was also expressed more in the male than in the female antennae, but this difference was limited to 3 times more. For the other OR genes tested, the *Bdoror16*, *Bdoror23*, *Bdoror8*, *Bdoror25*, *Bdoror22* and *Bdoror24* genes were expressed at similar levels in the antennae of the two sexes.

## Discussion

The oriental fruit fly is an important phytophagous pest and is considered a quarantine insect in many countries [36]. Although some plant-originated volatiles have been identified for the quarantine and control of this pest, few studies have examined the mechanism underlying chemical communication in *B*. *dorsalis*. The identification of the genes responsible for volatile perception is a prerequisite for the elucidation of the molecular mechanism of olfaction. Candidate olfaction genes have been identified in several other species through antennal transcriptome analysis and annotation [37–41]. However, *B*. *dorsalis* olfaction research has been limited by a lack of transcriptome data for the classical olfactory organ, namely the antennae, although several studies of the *B*. *dorsalis* transcriptome have recently been conducted [29–31]. To obtain general information about *B*. *dorsalis* olfaction, the transcriptomes of male and female antennae were sequenced and analyzed in the present study, and 20 OBPs, 5 CSPs, 35 ORs, 14 IRs and 4 SNMPs were successfully identified in the antennae. The 4 SNMP genes included 2 paralogs of SNMP1 and 2 paralogs of SNMP2. Paralogous genes have not been reported in *Bactrocera* and were previously limited to *C*. *quinquefasciatus* [42]. Although SNMP is required for pheromone detection [43], its function remains elusive because it is expressed in classical olfaction organs as well as in other tissues, including the head, legs and wings [44]. The annotation of *B*. *dorsalis* SNMP genes in this study may contribute to our understanding of the molecular function of SNMPs.

OBPs and CSPs, which are expressed at high concentrations in the sensillum lymph, are believed to assist in transporting organic semiochemicals across the aqueous sensillum lymph to the receptors (ORs or IRs) on the sensory neurons in the perireceptor process. Transcriptome analysis has become a useful method to identify the differentially expressed genes in a specific tissue or during different development stages of insects. Previous transcriptome analysis indicated that the number of OBPs ranged between 12–49 and the number of CSPs varied greatly from 4 to 22 in 15 insect species from Lepidoptera, Neuroptera, Coleoptera, Diptera and Homoptera [38,45–58]. We attribute this discrepancy to the diversity of expression of OBPs and CSPs in insect species, particularly OBPs and CSPs that are not specifically expressed in the antennae. In a previous study of *B*. *dorsalis*, 10 OBPs were identified in the transcriptomes of larvae, pupae and adults. Only 4 OBP genes identified in the antennal transcriptome were identical to previously reported OBP genes (*Bdorobp1*, *Bdorobp2*, *Bdorobp7*, *Bdorobp10*). Some OBP genes were expressed at levels that are too low to be detected in the RNA extracted from larvae, pupae and adults, thus suggesting that additional sequencing is required to further explore OBPs and CSPs via transcriptome.

Insect ORs play a key role in odor detection and discrimination after the release of semiochemicals from OBPs. However, no OR genes have been identified in *B*. *dorsalis*, with the exception of an Orco receptor gene that was also identified in the present study [9]. In this study, 35 OR candidate genes were identified, including 4 OR genes with relatively high sequence similarity to the *Drosophila* pheromone receptor *Dmor67d* (the respective identities of *Bdoror13*, *Bdoror35*, *Bdoror18* and *Bdoror16* to *Dmor67d* are 29.6%, 31.2%, 32.0% and 12.5%). Male-specific ORs potentially function in pheromone detection, whereas ORs expressed in the female antennae are predicted to function in oviposition-related odorant detection; ORs expressed in both the male and female antennae are predicted to function in general odorant perception [59–62]. In this study, the expression profiles of ORs and other olfaction genes were analyzed by comparing the transcript levels of the candidate genes in the different sexes. The OR gene biased expression patterns in *B*. *dorsalis* were similar to the biased expression pattern of those OR genes in other species, suggesting that the identified ORs may have functions similar to the functions of the ORs in other species [63]. There existed a differential expression between the male and female antennae for a selection of the candidate olfaction genes, including OR genes. The results were confirmed by qPCR analysis of nine randomly selected OR genes in different tissues. Interestingly, the two OR genes *Bdoror13* and *Bdoror14* showed a high and specific expression in the male antennae. In *Drosophila*, ORs play important roles in semiochemical (including pheromone) detection, which led us to study the potential of these proteins to explore new biomimetic odorant sensors in pest control. Thus, these ORs, which are assumed to be involved in olfaction in *B*. *dorsalis*, should also be explored in future research for potential control strategies.

IRs are critical in *D*. *melanogaster* chemical communication [64], however, this role remains to be confirmed in *B*. *dorsalis*. Here, we identified 14 IRs from the antennal transcriptome, which is far lower than the number of annotated IRs in mosquitoes and *D*. *melanogaster*. IRs are believed to function in oviposition and other behaviors, and thus they are likely not expressed in the antennae in this species [24, 65]. Among the identified IRs, the *Bdorir15*, *Bdorir16* and *Bdorir3* genes clustered with the co-receptor genes *Ir8a* and *Ir25*, and thus they are assumed to function in a similar manner [66]. Further studies in *B*. *dorsalis* are in progress based on the results of the present work.

## Conclusions

To explore the genes that are involved in olfactory signal detection and transduction in *B*. *dorsalis*, we determined here the transcriptome of the male and female antennae of oriental fruit fly using Illumina HiSeq 2000 sequencing technology. In total, 20 OBPs, 35 ORs, 14 IRs, 5 CSPs and 4 SNMPs were identified. Most of these genes were annotated for the first time in this study. These genes show a differential expression profile between males and females, indicating that they are involved in sex-specific olfaction. This information provides a foundation for future research on the molecular basis of the *B*. *dorsalis* olfactory system and for comparative and functional genomic analyses of other Diptera species.

## Supporting Information

S1 Table Primers used in the experiments. (DOCX)

S1 Text Information about the male and female *B*. *dorsalis* transcriptomes in GenBank. (TXT)

S2 Text FASTA format of the protein sequences of OBPs, CSPs, ORs, IRs, and SNMPs identified in this study. (TXT)
