# Supplementary material for: Identification of Male- and Female-Specific Olfaction Genes in Antennae of the Oriental Fruit Fly (Bactrocera dorsalis)
Source: PLoS One. 2016 Feb 4;11(2):e0147783. doi: 10.1371/journal.pone.0147783 (PMC4741523; doi:10.1371/journal.pone.0147783)
Supplement: S1 Table — (DOCX) [file pone.0147783.s001.docx]

S1 Table. Primers used in the qPCR experiments.

| Gene name | Direction^a^ | Nucleotide Sequence |
| --- | --- | --- |
| U33 | F | 5'-TGCCTTCAGCGTAATTTGTG-3' |
| U33 | R | 5'-GCCGATCTGCATGGTTATCT-3' |
| U350 | F | 5'-CGGTATTAACGCGTCCAACT-3' |
| U350 | R | 5'-GCTGCAAACCATACAGACGA-3' |
| U1586 | F | 5'-TGATCAATTTTGCGATGGAA-3' |
| U1586 | R | 5'-TTGAATTGCACGACCATCAT-3' |
| U8910 | F | 5'-TGAACGATTTCTGCGAAGTG-3' |
| U8910 | R | 5'-ATTTCTTGGCCGAATCAATG-3' |
| U4218 | F | 5'-CGCGCTCACATATCTTTTCA-3' |
| U4218 | R | 5'-GCCAGTAATTGGCTGTTGGT-3' |
| U11167 | F | 5'-CGATCCACCTCACTCCAAGT-3' |
| U11167 | R | 5'-GTGAGTGCAGCTCCGATGTA-3' |
| U8871 | F | 5'-GTGCTCGCGTGATCAAAATA-3' |
| U8871 | R | 5'-AATGACACGCCAAATCTTCC-3' |
| U10148 | F | 5'-GCATCTTAAGTCGCCTCGTC-3' |
| U10148 | R | 5'-TGTATTGCACGCCCATAAAA-3' |
| U3077 | F | 5'-TATACGATTTGCGCCAACAA-3' |
| U3077 | R | 5'-AATACCTACGCGGATGATGC-3' |

^a^ F: forward primer; R: reverse primer
